# Supplementary material for: Sustained-input switches for transcription factors and microRNAs are central building blocks of eukaryotic gene circuits
Source: Genome Biol. 2013 Aug 23;14(8):R85. doi: 10.1186/gb-2013-14-8-r85 (PMC4054853; doi:10.1186/gb-2013-14-8-r85)
Supplement: Additional file 5 — HTML Browsable Motif Output. Zipped folder containing all WaRSwap and FANMOD motif output, viewable in a web browser. [file gb-2013-14-8-r85-S5.ZIP › HTML_browsable_motif_output/FANMOD_ath_tair9/sigs_fanmodm-2000.pvals.heatmaps.html/motif_id_6_000101001_tftype_ath_upstream_-1000_0.html]

```
BG_MODEL = FANMOD
MOTIF_ID = 6_000101001
TF_TYPE = ath
UPSTREAM = -1000_0


PVals
FN_0.2	FN_0.4	FN_0.6	FN_0.8
dg_60.genes	0.643	0.764	0.488	0
dg_70.genes	0.629	0.766	0.488	0
dg_80.genes	0.621	0.749	0.526	0

ZScores
FN_0.2	FN_0.4	FN_0.6	FN_0.8
dg_60.genes	-0.409	-0.75	0.027	3.336
dg_70.genes	-0.369	-0.73	0.045	3.346
dg_80.genes	-0.337	-0.7	-0.06	3.29

StDevs
FN_0.2	FN_0.4	FN_0.6	FN_0.8
dg_60.genes	238.611	234.142	87.355	16.581
dg_70.genes	235.306	225.481	86.702	16.307
dg_80.genes	241.266	231.104	87.612	16.789
```
